# CDC Grand Rounds: Public Health Strategies to Prevent Neonatal Abstinence Syndrome

**DOI:** 10.15585/mmwr.mm6609a2

**Published:** 2017-03-10

**Authors:** Jean Y. Ko, Sara Wolicki, Wanda D. Barfield, Stephen W. Patrick, Cheryl S. Broussard, Kimberly A. Yonkers, Rebecca Naimon, John Iskander

**Affiliations:** ^1^Division of Reproductive Health, National Center for Chronic Disease Prevention and Health Promotion, CDC; ^2^Association of Schools and Programs of Public Health, Washington, D.C.; ^3^Office of the Associate Director for Science, CDC; ^4^Departments of Pediatrics and Health Policy, Division of Neonatology, Vanderbilt University School of Medicine, Nashville, Tennessee; ^5^Division of Congenital and Developmental Disorders, National Center on Birth Defects and Developmental Disabilities, CDC; ^6^Departments of Psychiatry, Obstetrics, Gynecology & Reproductive Sciences, Yale School of Medicine, New Haven, Connecticut.

## Public Health Burden of Neonatal Abstinence Syndrome

Neonatal abstinence syndrome (NAS) is a drug withdrawal syndrome that most commonly occurs in infants after in utero exposure to opioids, although other substances have also been associated with the syndrome (*1*). NAS usually appears within 48–72 hours of birth with a constellation of clinical signs, including central nervous system irritability (e.g., tremors), gastrointestinal dysfunction (e.g., feeding difficulties), and temperature instability (*1*) (Box 1). Opioid exposure during pregnancy might result from clinician-approved use of prescription opioids for pain relief; misuse or abuse of prescription opioids; illicit use (e.g., heroin); or medication-assisted treatment (MAT) of opioid use disorder (*2*) (Box 2).

BOX 1 **Signs of neonatal abstinence syndrome**

**Central nervous system irritability**

- High-pitched, continuous crying
- Decreased sleep
- Tremors
- Increased muscle tone
- Hyperactive Moro reflex
- Seizures
- Gastrointestinal dysfunction
- Feeding difficulties
- Vomiting
- Loose/watery stools

**Autonomic nervous system activation**

- Sweating
- Fever
- Frequent yawning and sneezing
- Increased respiratory rate
- Nasal stuffiness and flaring

BOX 2 **Opioid exposures associated with neonatal abstinence syndrome**

- Prescription pain relievers, such as Vicodin, OxyContin, Percocet
- Illicit substances: heroin or nonpharmaceutical formulations of fentanyl
- Opioid-assisted maintenance therapy (also known as medication-assisted treatment): long-term treatment for opioid use disorder, under medical supervision, with a longer-acting but less euphoric opioid
  - Methadone or buprenorphine is recommended by the American College of Obstetricians and Gynecologists during pregnancy

Opioid pain reliever sales quadrupled in the United States from 1999 to 2010. From 2000 to 2014, opioid-related overdoses among U.S. adults increased 200% (*3*). Opioid use during pregnancy has also increased nationally in recent years. The percentage of Medicaid-enrolled women who filled at least one opioid prescription during pregnancy increased 23% during 2000–2010, from 18.5% to 22.8% (*4*). The prevalence of opioid abuse or dependence among pregnant women has increased from 1.7 per 1,000 delivery admissions in 1998 to 3.9 in 2011 (*5*). Reflective of increasing maternal opioid exposure, the incidence of NAS has increased approximately 400% nationally, from 1.2 per 1,000 hospital births in 2000 to 5.8 in 2012, with some states reporting rates in excess of 30 per 1,000 hospital births (*6*,*7*). By 2012, on average, one NAS-affected infant was born every 25 minutes in the United States (*6*).

Respiratory and feeding difficulties, low birth weight, and seizures are more prevalent among infants with NAS (*1*). Care for infants with NAS has placed a substantial burden on hospitals, particularly on neonatal intensive care units. In 2012, a term infant without complications had a mean length of stay of 2.1 days and charge of $3,500; whereas, an infant with NAS had a mean hospital stay of 16.9 days and a mean hospital charge of $66,700 (*6*). Aggregate hospital charges for all infants with NAS in 2012 were estimated to be $1.5 billion; approximately 80% was financed by Medicaid programs (*6*). Public health measures to prevent and treat opioid dependence before and during pregnancy are essential to reducing the incidence of NAS and its related health care burden. Strategies include promoting responsible opioid prescribing, decreasing unplanned pregnancies among women who abuse opioids, screening and treatment during pregnancy, and standardizing postnatal treatment for infants with NAS (Figure).

**FIGURE:**
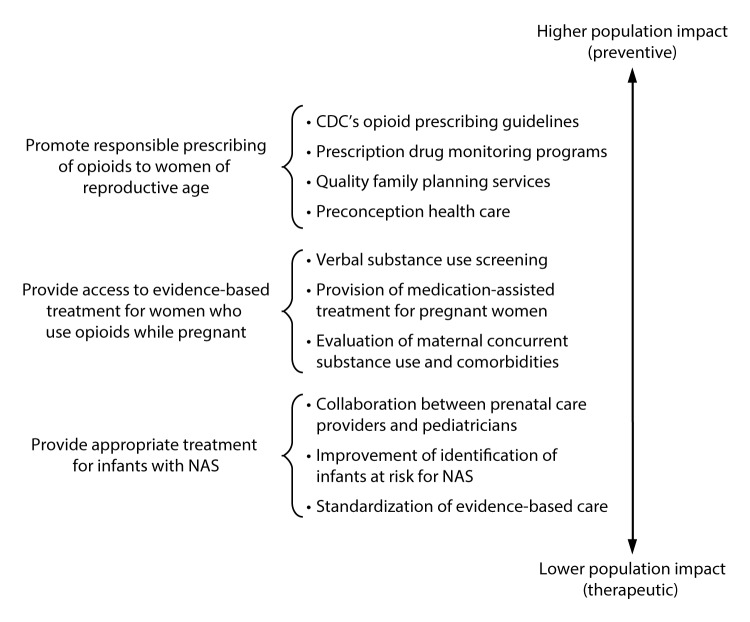
Specific strategies to reduce the health care burden of neonatal abstinence syndrome (NAS), by population impact

## Prevention and Intervention Strategies

**Primary prevention.** Strategies to prevent the incidence of NAS center on responsible opioid prescribing and access to preconception care and family planning services (Figure). The 2016 *CDC Guideline for Prescribing Opioids for Chronic Pain* (*8*) recommends that clinicians address the unique sensitivities of prescribing opioid medications to pregnant women and nonpregnant women of reproductive age. Recommended actions include discussing how long-term opioid use might affect current and future pregnancies and how women of reproductive age with a need for long-term opioid use can avoid unintended pregnancy. Clinicians and patients together should carefully weigh risks and benefits when deciding whether to initiate opioid therapy for chronic pain during pregnancy. Other specific recommendations for women of reproductive age include considering nonopioid pharmacologic therapy for chronic pain management and prescribing the lowest effective dose when opioids are started. As of March 2016, the Food and Drug Administration requires both sustained and immediate-release opioid medication to include a black box warning, informing patients that prolonged opioid use during pregnancy might lead to NAS.

Prescription drug monitoring programs (PDMPs) are an important public health tool to support responsible opioid prescribing. PDMPs are state-based databases that track controlled prescription drugs dispensed by pharmacies and allow prescribers to review a patient’s prescription history before prescribing. Every state except Missouri has implemented a PDMP (*9*). Preliminary research suggests that PDMPs have been associated with a reduction in opioid-related deaths in the general population (*9*). Successful application of PDMPs requires that prescribing health care providers are both aware of and regularly use the PDMP database (*9*). However, barriers, such as provider time constraints and lack of data integration into electronic medical records, might prevent universal adoption of PDMPs by health care providers. CDC continues to fund states for prescription drug overdose prevention activities, including maximizing PDMP use and improving timeliness of data availability.*

Another primary prevention strategy that might reduce the incidence of NAS is ensuring access to family planning and preconception care for women who use opioids. Among women who abuse opioids, 86% of pregnancies are unintended (*10*). CDC and the Office of Population Affairs of the U.S. Department of Health and Human Services recommend that health care providers support family planning services, which include preconception services, pregnancy intention screening, and contraceptive counseling to prevent unintended pregnancy by increasing access to the full range of contraceptive methods, including long-acting reversible contraception (e.g., intrauterine devices and implants) (*11*).

Two national initiatives encourage clinicians to practice responsible prescribing and help women of reproductive age optimize their health before pregnancy. CDC’s Treating for Two: Safer Medication Use in Pregnancy initiative^†^ encourages evidence-based prescribing practices and informed decision-making specifically for pregnant women and for nonpregnant women of reproductive age. The second, the National Preconception Health and Health Care Initiative,^§^ provides educational resources to clinicians and their patients, and coordinates outreach and social media campaigns related to improving preconception health, including reducing substance use and treating substance use disorders before pregnancy.

**Intervention strategies and treatment for women with opioid use disorder**. Provision of treatment for pregnant women with opioid use disorder is important. Medically supervised tapering of opioids in pregnant women is associated with high relapse rates as compared to methadone maintenance (*2*). The Substance Abuse and Mental Health Services Administration (SAMHSA) and the American College of Obstetricians and Gynecologists recommend that pregnant women with opioid use disorder receive MAT with methadone or buprenorphine (*2*,*12*). SAMHSA’s Substance Abuse Prevention and Treatment block grants have recently been revised to strengthen capacity to deliver MAT for pregnant women with substance use disorders. It is important that clinical providers evaluate concurrent substance use (e.g., tobacco) and common maternal psychiatric (e.g., depression) and infectious (e.g., hepatitis C) comorbidities of opioid use disorder (*13*). In addition, clinical providers should anticipate that infants born to women receiving MAT might have NAS (*8*). Collaboration with pediatric care teams is necessary to assess infants with in utero opioid exposure for signs of NAS and provide appropriate treatment.

**Strategies to mitigate the effects of NAS.** Improvements in the identification of infants at risk and standardized treatment of infants with NAS could greatly mitigate the effects of NAS and the associated health care burden. To enable state health departments identify and provide interventions to areas with high NAS incidence, four states (Florida, Georgia, Kentucky, and Tennessee) have designated NAS a reportable condition.

Treatment of NAS begins with nonpharmacological strategies, such as minimizing environmental stimuli (e.g., placement in a dark, quiet space), careful swaddling, and, in the absence of other contraindications, breastfeeding. In addition, severe NAS requires tapered dosages of morphine or methadone as recommended by the American Academy of Pediatrics, to ease infants’ withdrawal, coupled with nonpharmacologic strategies (*1*). Further, recent studies have found that hospital-level strategies, including rooming-in, rather than infant placement in neonatal intensive care units, increased family involvement during hospitalization (*14*), and standardized opioid-weaning guidelines (*15*), were associated with shorter lengths of hospital stay for infants with NAS. A multicenter quality improvement study aimed at standardization of hospital care for infants with NAS demonstrated that implementation of standardized procedures for assessing infants at risk and treating infants with NAS decreased both their length of hospital stay and need for pharmacological treatment (*16*). State perinatal quality collaboratives are networks of perinatal care providers and public health professionals working together to advance evidence-based clinical practices and processes through quality improvement. Quality improvement projects addressing the care of infants with NAS are ongoing.^¶^

**Barriers to prevention and treatment.** As a part of complete obstetric care, The American College of Obstetricians and Gynecologists recommends that all pregnant women be routinely asked about their substance use, including prescription opioids and other medications used for nonmedical reasons (*2*). Despite the importance of detecting substance use disorders among pregnant women to offer timely treatment, there is little consensus regarding the best screening tool to identify substance use among pregnant women, the best time during pregnancy to screen, and whether biologic specimens should be used in conjunction with or in lieu of verbal screening. To address the need for reliable substance use screening during pregnancy, CDC is currently funding a three-site research study to assess the performance of five different substance use screening tools for pregnant women (NIH RePORT Project number: 5R21DP006082–02).

Stigma, provider bias, and legal consequences pose additional barriers to screening and subsequent identification of women in need of treatment. Screening for substance abuse during pregnancy and compliance with legally mandated reporting might be subject to provider bias in contrast to adherence to objective medical criteria (*17*). Furthermore, legal ramifications for maternal substance use vary by state. Eighteen states classify maternal substance use as child abuse and three states consider it grounds for involuntary hospitalization.** Conversely, 19 states provide specialized drug treatment programs for pregnant women, 13 states prioritize pregnant women in state-funded drug treatment programs, and four states legally prohibit discrimination against pregnant women who seek substance abuse treatment. The impacts of these varied state legislations on prenatal care attendance, disclosure of substance use, and treatment seeking or receipt are unclear.

## Federal Legislation and Awareness

Two recent legislative initiatives and a nationwide call to action address maternal opioid use and NAS. The Protecting Our Infants Act of 2015^††^ stipulates that the U.S. Department of Health and Human Services conduct a review of intra-agency work related to NAS and prenatal opioid exposure to identify gaps or overlap in research or programs and to provide technical assistance to states and American Indian tribes when implementing public health measures, including NAS surveillance systems. The Comprehensive Addiction and Recovery Act of 2016^§§^ extends the federal grant program for state-based PDMPs and support for various substance use disorder treatment services for pregnant women and infants.

In addition, the U.S. Surgeon General’s Turn the Tide campaign^¶¶^ calls for clinicians to treat pain safely and effectively, screen patients for opioid use disorder, and provide or connect them with evidence-based treatment, and recognize and treat addiction as a chronic illness, and not a moral failing.

## Conclusion

NAS is an often hidden consequence of the opioid epidemic. The incidence of NAS has increased sharply over the last decade and is associated with substantial health care expenditures. Responsible opioid prescribing practices, including use of PDMPs, and increased availability of preconception health services are vital primary prevention strategies. Access to treatment for maternal opioid use disorder and standardized treatment for infants with NAS might further decrease the effects of NAS.

## Footnotes

* https://www.cdc.gov/drugoverdose/states/state_prevention.html.

† https://www.cdc.gov/pregnancy/meds/treatingfortwo/.

§ https://beforeandbeyond.org/.

¶ https://opqc.net/projects/NAS.

** https://www.guttmacher.org/state-policy/explore/substance-abuse-during-pregnancy.

†† https://www.congress.gov/bill/114th-congress/senate-bill/799.

§§ https://www.congress.gov/bill/114th-congress/senate-bill/524/text.

¶¶ http://turnthetiderx.org/.
